# The effect of bariatric surgery on female sexual function: a cross-sectional study

**DOI:** 10.1038/s41598-020-69176-8

**Published:** 2020-07-22

**Authors:** Anna Różańska-Walędziak, Paweł Bartnik, Joanna Kacperczyk-Bartnik, Maciej Walędziak, Andrzej Kwiatkowski, Krzysztof Czajkowski

**Affiliations:** 1 2nd Department of Obstetrics and Gynecology, Medical University of Warsaw, Karowa 2 St., 00-315 Warsaw, Poland; 2 Department of General, Oncological, Metabolic and Thoracic Surgery, Military Institute of Medicine, Szaserów 128 St., 04-141 Warsaw, Poland

**Keywords:** Diseases, Medical research

## Abstract

The generally negative impact of obesity on female sexuality is well-established. The possible association between bariatric surgery, weight loss, and female sexuality is much less described. The aim of the study was to analyse the possible association between bariatric surgery and female sexual function. It was a cross-sectional study of 623 patients who underwent bariatric surgery between 1999 and 2017. Patients were recruited on the basis of medical records from the Military Institute of Medicine in Warsaw. Patients were invited to complete a questionnaire which consisted of self-designed demographic questions and Female Sexual Function Index (FSFI). The total FSFI score, as well as each subdomain, improved significantly after surgery. The prevalence of low score (< 26.55) was significantly lower after the surgery in comparison to the status prior to the procedure (36.3% vs. 57.5%; *p* < .001). There were no differences regarding the number of sexually active patients before and after the surgery (75.3% vs. 76.1%; *p* < .63). There were observed statistically significant, positive correlations between BMI decrease and each subdomain of the FSFI score as well as the total score. Weight loss surgery seems to decrease the risk of sexual dysfunction presence and the advantages are associated with the total BMI loss.

## Introduction

The prevalence of obesity among women in Europe and Poland was steadily increasing during the first 2 decades of the XXI century^1^. Obesity is associated with an increased risk of a number of chronic diseases, including diabetes mellitus, hypertension, sleep apnoea, and coronary heart disease^2^. In addition, it has a negative impact on the overall quality of life^3^.

The generally negative impact of obesity on female sexuality is well-established^4^. There is an inverse association between BMI and sexual functioning , including sexual dysfunction occurrence^5^. Sexual dysfunction is usually defined as persistent, recurrent problems with sexual response, desire, orgasm or pain that distress the patient or affect her relationship^6^. In the group of women who seek bariatric surgery, the prevalence of sexual dysfunction may be even higher^7^. The possible association between bariatric surgery, weight loss, and female sexuality is much less described. Whereas the positive impact of weight loss on the general well-being may be decisive, the loss of a large volume of hormonally-active fat tissue and drastic hormonal changes it implies may have a significant role too^8,9^

There are many ways of obesity treatment, one of which is bariatric (weight-loss) surgery. It includes various surgical procedures, such as sleeve gastrectomy, Roux-en-Y gastric bypass or adjustable gastric band^2^. Bariatric surgery is considered to be the most effective and decisive method of obesity treatment^10^. It is, however, associated with long-lasting consequences for the patients. Most patients require lifelong microelements supplementation and most techniques are irreversible^2^.

The aim of the study was to analyse the possible association between bariatric surgery and female sexual function.

## Results

The group characteristics are presented in Table 1. The mean age at the point of questionnaire completion was 39.3 ± 9.1 years. The median time from surgery to survey completion was 29.3 months. 85.6% of the respondents were in the pre-menopausal age and 95.7% were heterosexual. The majority, 65.6% of the patients underwent sleeve gastrectomy, following 26.3% underwent Roux-en-Y gastric bypass. The mean percentage excess weight loss (%EWL) was 72.9 ± 30.4%, and 80.4% of the patients reached the clinical goal of < 50%EWL.

**Table 1:** Group characteristics.

Feature (n = 623)	Value
Age (at interview)	39.3 ± 9.1
Time from surgery (Median; 1-3rd quartile) [months]	29.3; 18.8–45.5
Pre-surgery BMI (Mean; ± SD) [kg/m^2^]	41.2 ± 7.5
Pre-surgery weight (Mean; ± SD) [kg]	121.1 ± 20.3
Current BMI (Mean; ± SD) [kg/m^2^]	29.3 ± 6.3
Current weight (Mean; ± SD) [kg]	85.6 ± 18.3
BMI loss (Mean; ± SD) [kg/m^2^]	11.9 ± 6.1
Weight loss (Mean; ± SD) [kg]	34.9 ± 18.0
Currently smoking (%)	127 (20.4)
Post-menopausal	90 (14.4)
In a relationship (%)	512 (82.8)
Sexual orientation (%)	
Heterosexual	579 (95.7)
Homosexual	9 (1.5)
Bisexual	16 (2.6)
Type of surgery (%)	
Sleeve gastrectomy	403 (65.6)
Roux-en-Y gastric bypass	164 (26.3)
Sleeve gastrectomy + Roux-en-Y gastric bypass	17 (2.7)
Adjustable gastric band	14 (2.2)
Does not know/uncertain	26 (4.1)
Excess weight loss (Mean ± SD; %)	72.9% ± 30.4
Patients who achieved 50% excess weight loss (%)	495 (80.4%)
Comorbidities (%)	
Diabetes (any form)	156 (25.0)
Hypertension	133 (21.3)
Sleep apnoea	17 (2.7)
Hypothyroidism	130 (20.8)
Coronary heart disease	5 (0.8)

The comparison of FSFI scores before and after the surgery are presented in Table 2. The total FSFI score, as well as each subdomain, improved significantly after surgery. The prevalence of low score (< 26.55) was significantly lower after the surgery in comparison to the status prior the procedure (36.3% vs. 57.5%; *p* < 0.001). There were no differences regarding the number of sexually active patients before and after the surgery (75.3% vs. 76.1%; *p* < 0.63).

**Table 2:** FSFI results.

Feature	Preoperative	Postoperative	*p*
Desire	3.2 ± 1.3	3.8 ± 1.2	< .001
Arousal	3.6 ± 1.5	4.4 ± 1.5	< .001
Lubrication	4.2 ± 2.1	4.5 ± 1.9	< .003
Orgasm	3.7 ± 1.9	4.3 ± 1.9	< .001
Satisfaction	3.7 ± 2.0	4.4 ± 2.0	< .001
Pain	4.1 ± 1.9	4.5 ± 1.9	< .001
Total	22.3 ± 9.5	25.9 ± 9.4	< .001
Score < 26.55 occurrence (%)	318 (57.5)	201 (36.3)	< .001
Sexually active (%)	470 (75.3)	475 (76.1)	.64

The Spearman linear correlation between FSFI score change as well as all of its subdomains separately and continuous demographic parameters are presented in Table 3. There were observed statistically significant, positive correlations between BMI decrease and each subdomain of FSFI score as well as a total score. Negative correlations between age and each subdomain of FSFI, with the exception of the desire subdomain, were observed.

**Table 3:** Correlations between changes in the FSFI score and demographic parameters.^1^

	BMI pre	BMI post	BMI change	%EWL	Age	Months from surgery
Desire (change)	.03 .56	− .08 .07	**.12** **.005**	**.10** **.02**	− .03 .54	− .02 .62
Arousal (change)	.05 .25	**− .09** **.03**	**.17** **.001**	**.14** **.001**	**− .11** **.008**	.03 .48
Lubrication (change)	.06 .17	− .08 .054	**.18** **.001**	.06 .18	**− .09** **.03**	.03 .38
Orgasm (change)	.08 .06	− .08 .07	**.20** **.001**	**.11** **.01**	**− .13** **.003**	.01 .83
Satisfaction (change)	**.10** **.02**	− .05 .23	**.20** **.001**	**.09** **.03**	**− .13** **.003**	− .01 .74
Pain (change)	**.11** **.01**	− .04 .31	**.20** **.001**	.07 .10	**− .10** **.03**	− .01 .88
Total (change)	.08 .06	− .08 .07	**.20** **.001**	.08 .06	**− .11** **.01**	.01 .86

^1^Cell organization: upper value–R; lower value–p; bold values‐statistically significant.

## Discussion

Analysis of FSFI results from our patients revealed significant improvement in the general sexual function as well as in each subdomain. It is an interesting observation, which stays in coherence with other studies which used similar methodology^4,9–16^. Only a study by Luyssen et al. on a relatively small group of patients (n = 71) showed no significant change in sexual functioning after weight loss surgery^17^. There was observed a statistically significant decrease in the prevalence of FSFI score lower than 26.55 from 57.3% to 26.3%. It is an important and significant observation, as on one hand, it has a clinical significance – the shift from being at high risk of sexual dysfunction to being at low risk is highly desirable. No study with this approach was found in the literature.

Surprisingly, no significant change in the number of sexually active patients was observed in the analysed group. Approximately 3 quarters of patients were sexually active before and after weight loss surgery. As we observed the improvement in the FSFI score we expected at least some increase in sexually active patients, as those two variables usually correlate^18^. Leshem et al. and Treacy et al. made a similar observation^14,16^. Conason et al. observed no differences in the frequency of sexual activity in a 2-year follow-up^13^.

There were observed statistically significant positive correlations between the BMI decrease and the FSFI score, both total and in all of its subdomains. Moreover, we observed similar, statistically significant correlations between %EWL and desire, arousal, orgasm and satisfaction FSFI subscales. Similar, but slightly stronger correlations, using the same tool (FSFI) were observed by Assimakopoulos et al.^19^. In addition to this, there were observed two positive correlations between preoperative BMI and satisfaction and pain subdomains changes. A negative correlation was observed between postoperative BMI and improvement in the arousal subscale. The previous study by Janik et al., performed on a smaller cohort of patients showed a moderate, negative correlation between total FSFI score and postoperative BMI^20^. In this study, we decided to focus on the practical, clinical aspects of the issue. Therefore it seems that the feature which has the strongest association with sexual function improvement is neither with the BMI prior to the surgery, nor the BMI obtained after surgery, but the BMI improvement/EWL lost.

Negative correlations were observed between age and FSFI score change, in all of its domains with the exception of the desire subdomain. We have not found another study with a similar observation. It seems that with increased age the sexual function benefit obtained from bariatric surgery decreases, or on the other hand, the younger patients benefit from it more.

The main source of bias in this study results from its cross-sectional nature. The fact that patients answer both questions, about the preoperative sexual functioning and their current status may lead to the recall bias. The lack of face-to-face medical interview limits the possibility of female sexual dysfunction as it is essential for the diagnosis in addition to the raw FSFI score. Therefore we decided to use term “score lower than 26.55” rather than “female sexual dysfunction”. It seem that future studies should include direct, face-to-face interview with sexologist before the surgery and after a certain period of time in order to eliminate problems this study experienced.

The improvement of sexual function is a certain additional benefit from bariatric surgery. Weight loss surgery seems to decrease risk of sexual dysfunction presence and the advantages are associated with the total BMI loss. Younger age may predispose to higher increase in sexual function quality resulting from bariatric surgery.

## Methods

It was a cross-sectional study of 623 patients who underwent at least one bariatric surgery between 1999 and 2017. It was a part of a project analysing the effects of bariatric surgery on the course of pregnancy. The study was performed in accordance with the ethical standards as laid down in the 1964 Declaration of Helsinki and its later amendments. Participants were informed about the aim of the study, and informed consent was obtained electronically prior to the beginning of the survey. The approval from Military Institute of Medicine Ethics Committee was obtained on 22nd August 2018 (no. 117/WIM/2018).

Patients were recruited on the basis of medical records from the Military Institute of Medicine in Warsaw. All patients underwent the sleeve gastrectomy (SG), Roux-en-Y gastric bypass (RYGB) or adjustable gastric band (AGB). Some of the patients underwent both SG and RYGB. All participants met the Interdisciplinary European Guidelines on Metabolic and Bariatric Surgery criteria for bariatric surgery^21^. Each patient was contacted via telephone and invited to complete a questionnaire which consisted of self-designed demographic questions, questions about pregnancies which occurred after the surgery (presented elsewhere) and a polish version of the Female Sexual Function Index (FSFI)^22^. Each patient was contacted at least 3 times in the 48-h period before considered unapproachable. Patients completed the questionnaires anonymously via an online questionnaire, paper version of the questionnaire or through a telephone conversation with a medical doctor. The method of questionnaire completion depended on patient’s preferences.

**Figure 1:**
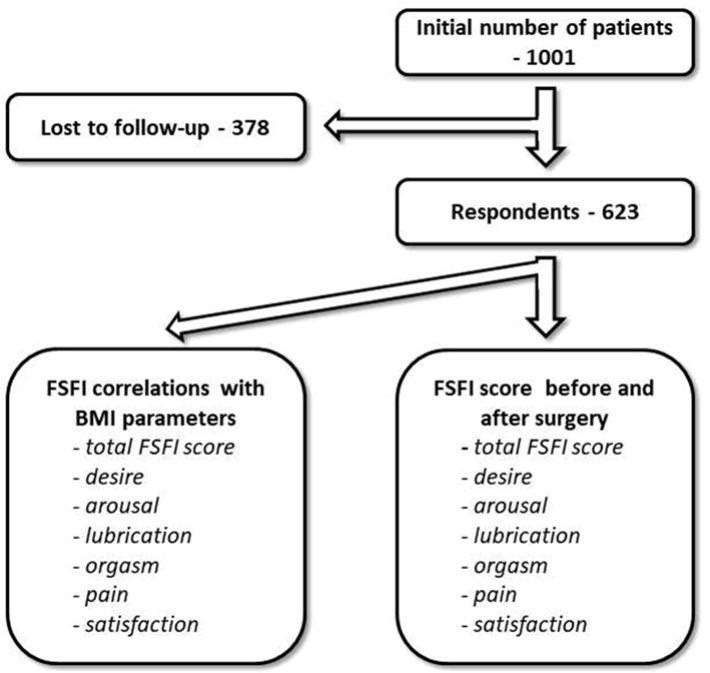
Study design. The number of contacted patients was 1,001. 623 women completed the questionnaire. Each patient was contacted via telephone and invited to complete a questionnaire including the Polish version of the Female Sexual Function Index (FSFI), which consists of 19 questions, analysing the spheres of desire, arousal, lubrication, orgasm, pain, and satisfaction. At the point of questionnaire completion, patients were asked about last month sexual performance and about the last month before the surgery. Correlations between BMI decrease and each subdomain of FSFI score were also investigated.

The initial number of patients contacted was 1,001, among which 623 completed the questionnaire. The response ratio was 62.3%. Study design is presented in Fig. 1.

The Female Sexual Function Index is one of the most widely-used, standardised questionnaires used to assess sexual function in women^17^. It was translated to Polish and validated^21^. It consists of 19 questions, which separately analyse the spheres of desire, arousal, lubrication, orgasm, satisfaction, and pain. The maximum score is 40 and the minimum is 2 points. The score lower than 26.55 indicates a high risk of female sexual dysfunction (FSD) occurrence. At the point of questionnaire completion, patients were asked about last month sexual performance and about the last month before the surgery.

Statistical analysis was performed using Statistica 13 (StatSoft. Inc.; English version). U-Mann Whitney test and *t* Student tests were used for quantitative data comparison between two groups as required. Spearman correlation coefficient was applied for linear correlation between two variables. Two-sided Fisher’s exact test and chi-square test were used for categorical and binary data comparison as required. *p* value < 0.05 was considered significant.
